# Laccase Enzyme Polymerization by Soft Plasma Jet for Durable Bioactive Coatings

**DOI:** 10.3390/polym10050532

**Published:** 2018-05-16

**Authors:** Szymon Malinowski, P. Anthony F. Herbert, Jerzy Rogalski, Justyna Jaroszyńska-Wolińska

**Affiliations:** 1 Civil Engineering and Architecture Faculty, Lublin University of Technology, 20-618 Lublin, Poland; s.malinowski@pollub.pl (S.M.); j.wolinska@gmail.com (J.J.-W.); 2 Plasma Ireland, T23 N592 Cork, Ireland; 3 Department of Biochemistry, Maria Curie-Skłodowska University, 20-031 Lublin, Poland; Rogal@poczta.umcs.lublin.pl

**Keywords:** bioactive coatings, plasma deposition, laccase, soft plasma polymerization

## Abstract

Conventional pin-to-point continuous wave Helium Corona plasma discharge was successfully used in Soft Plasma Polymerization (SPP) processes to immobilize into water and onto glass polymerized bioactive *Cerrena unicolor* laccase coatings. The coatings were tested for bioactivity and durability under water wash. The coatings showed up to 59% bioactivity relative to the native laccase in water deposition, undoubtedly due to damage to and fragmentation of monomer molecules by the active, energetic species in the plasma. However, plasma deposited laccase coatings on glass delivered 7 times the laccase activity of the same non-plasma deposition process in the coating after water wash. This latter result would seem to be due to the ability of the plasma to both crosslink monomer and more strongly bond it to the glass surface by a combination of surface cleaning and the creation of active, high energy sites in both glass and laccase molecules. FTIR analysis indicated that the core copper containing moieties at the centre of the molecule largely remain undamaged by this plasma type so that bonding and cross-linking reactions are likely to mainly involve species around the outer perimeter of the molecule. The chemical composition and structure of laccase biocoatings deposited by Corona SPP are described. The combination of the coating performance parameter values for retained activity and durability under water wash indicates that a relatively simple Corona plasma process for deposition of biocoatings, which directly polymerizes the monomer with no added matrix or encapsulant material, may offer enhanced solutions for biocatalyst, sensor or lab-on-a-chip applications.

## 1. Introduction

Plasma techniques have become increasingly popular as surface engineering tools in recent years, in particular for deposition of functional coatings. Currently, plasma techniques are being developed because of the opportunity for deposition of coatings without using toxic solvents, thus being more eco-friendly than conventional methods [1]. A significant step in film deposition has been the development of atmospheric pressure, room temperature Soft Plasma Polymerization (SPP) processes. SPP is the plasma deposition of organic compounds where the original monomer is put down as a cross-linked, thin-film coating without major degradation or denaturing of the original monomer, namely without losing significant functional groups from the molecule [2]. The mechanism of SPP is complex and includes reactions both in the gas/plasma phase and on the surface of the solid substrate receiving the coating. These processes have been studied on non-bioactive organic precursors [3], where interaction between energetic electrons in the plasma and monomer precursor molecules generates radicals of the organic precursor. Radical-radical and radical-molecule interactions with participation of unsaturated bonds (double and triple) occur causing cross-linking between original monomer molecules and thus generation of polymerized organic molecules with a significantly higher molecular weight (MW) [4]. Examples of successfully plasma polymerized organic precursors are organosilicon compounds such as: TMCTS (tetramethylcyclosiloxane) [5], HMDSO (hexamethyldisiloxane) [6], HMDSN (hexamethyldisilazane) [7], TEOS (tetraethoxysilane) and TMDSO (tetramethylodisiloxane) [8].

Polymers formed in plasma reactions are characterized by a higher degree of cross-linking in comparison to polymers obtained by conventional methods, such as wet chemistry. The properties of plasma deposited polymeric coatings are significantly impacted by the energy density (J/mm^3^) of the plasma discharge, the type of carrier gas (inert such as He, Ar, Ne in simple SPP, chemically reactive such as O_2_ in Chemically Assisted SPP (CASPP)) and the type of solid substrate used in the process [3,4]. The simple SPP technique used in this work is based on atmospheric pressure, room temperature, non-equilibrium plasma jet in the form of a pin-to-plane Helium Corona discharge into which the precursor monomer is injected largely as vapor as opposed to a total aerosol. Aerosol monomer injection to achieve SPP has been employed [9] but it carries operational issues such as a rapid buildup of unwanted deposits on equipment inhibiting industrial application. This Corona discharge jet technique combines vapor monomer injection and low specific energy (J/mm^3^) plasma with fast, non-thermal equilibrium electrons, causing precursor polymerization without excessive damage to the monomer molecule. For example, in earlier research, this SPP technique was used for the deposition of HDFDA (*1H*, 1*H*, 2*H*, 2*H*-heptadecafluorodecyl acrylate). XPS and FTIR studies showed that polymerization of the organic compound occurred through participation of vinyl groups without excessive precursor fragmentation [2].

Plasma has been used both to prepare device surfaces for bioprocessing and to deposit what the literature often refers to as biofunctional coatings. These plasma deposited coatings, however, frequently do not contain bioactive molecules but only prepare the surface with ceramic or polymer coatings designed to accept the actual bioactive species, and are known as interlayers [10,11,12]. Application of these interlayers allows the introduction of functional groups, e.g., amine [13,14,15,16,17], carboxyl [18] or aldehyde, which are chemically active during the biocoating immobilization process [11]. Moreover, plasma treatment is applied to polystyrene film to improve enzyme bonding efficiency through surface activation, i.e., raising surface energy [19]. Interlayer coatings are used in bio-sensing as matrices for biomolecule immobilization [1]. The role of plasmas in biomolecule immobilization has to date been very limited because of their general inability to handle large, high molecular weight molecules without damage or destruction [10]. However, the literature has noted plasma procedures with organic polymer precursors for glucose oxidase, lipase and alkaline phosphatase immobilization onto glass slides and plastic foil [20]. We believe that this work using low specific energy Corona type plasma to achieve SPP significantly extends the capability of plasma-based enzyme immobilization. Sensors based on this coating technology are currently on trial.

The purpose of this work is to extend application of the SPP technique to the deposition of biologically active coatings. These studies investigate the impact of room temperature Helium Corona SPP on enzyme protein (laccase) and optimization of the plasma reaction conditions to achieve two essential functional coating properties as responses. These responses are cross-linking and bonding to the substrate surface to obtain a durable coating that will not simply wash away in water or solvent and retention of the biological functionality of the precursor, i.e., prevention of biomolecule denaturing. The combination of these two properties is essential to obtaining a meaningful bioactive functional coating on a substrate which can potentially be useful in applications such as sensors or lab-on-a-chip. This SPP process should be distinguished from plasma deposition of coatings comprising bioactive species embedded within a matrix, often called biocomposite coatings [21]. These consist of an organic matrix, e.g., ethylene, embedding a bioactive molecule. In such coatings there is no substantial polymerization of the active monomer. Instead, monomer molecules are injected into the plasma zone not in an inert carrier gas such as Helium, as with simple SPP, but in a chemically reactive carrier gas such as C_2_H_4_ ethylene. It is generally the reactive carrier that polymerizes into a coating while encapsulating the monomer molecules, which are individually distributed throughout the coating volume. Such coatings will invariably have different properties to coatings put down by simple SPP, although whether the differences are significant is entirely dependent upon the intended application.

Biocatalyzers (enzymes) are widely used in industrial procedures, replacing traditional methods and reducing the cost of production. These enzymes mainly belong to the oxidoreductase and hydrolase classes and are used in both native and immobilized forms [22], the latter being where the enzyme is attached to a substrate or matrix support. In recent years enzymes have grown in significance for production of biofuels such as biodiesel, bioethanol, or fuel cells [23]. However, these desirable characteristics of enzymes and their widespread industrial applications are often obstructed by their lack of long-term operational stability, shelf life and by their poor recovery and reusability. Enzyme immobilization is one of the strategies tried to overcome these problems. Immobilization of enzymes can be carried out by different methods broadly classified as physical and chemical. Physical methods have weak interactions and adhesion between matrix and enzyme, whereas in chemical methods there is formation of covalent bonding between the support and the enzyme. In particular, the development and applications of site selective protein immobilization has undergone significant advances in recent years. Advances in organic chemistry and molecular biology have led to the development of some very powerful, efficient, site-specific and important techniques for anchoring proteins onto supports [23,24].

**Figure 1:**
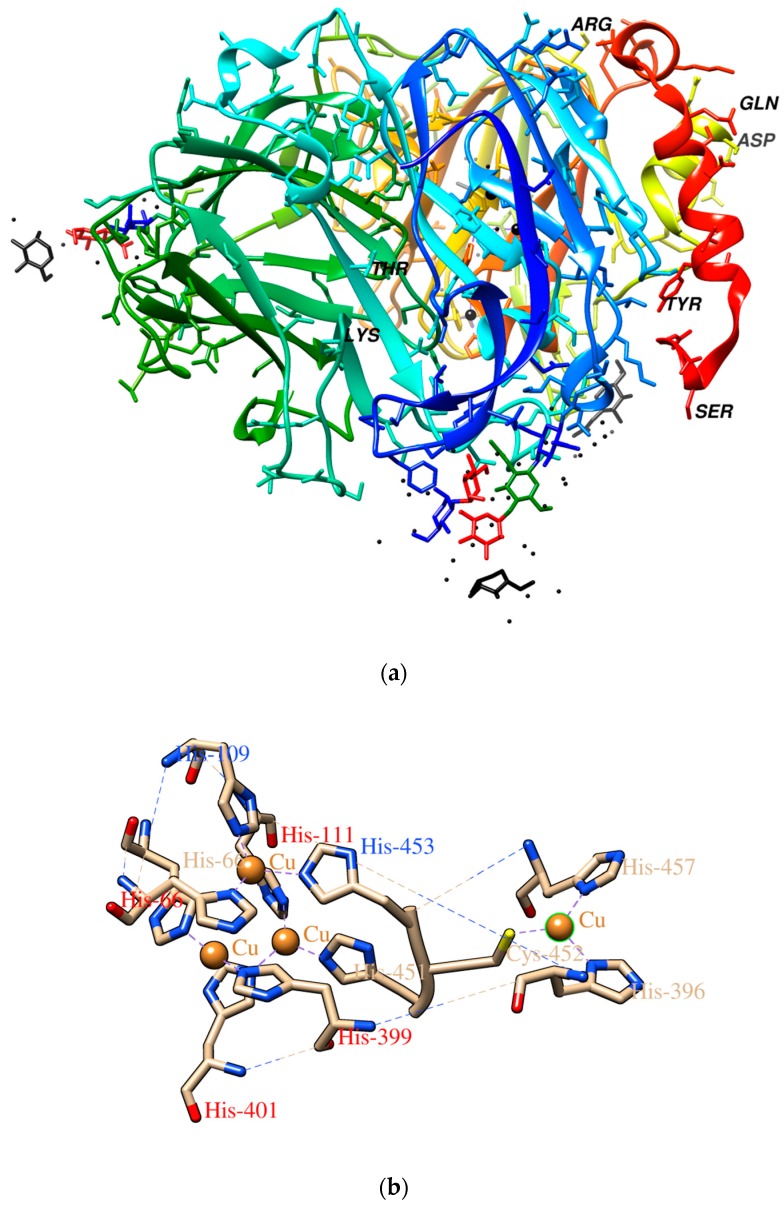
(**a**) 3D modelled structure of *Trametes versicolor* laccase [31] (**b**) and schematic representation of its copper coordination center [32].

The enzyme used in this work, laccase, belongs to the oxidoreductase class of enzymes synthesized by plants, fungi, some bacteria and insects. The active center of the laccase named blue oxidase includes 4 Cu (II) atoms divided into 3 parts labelled T1, T2 and T3 [25]. The three-dimensional structure of this enzyme molecule and its active center is shown in Figure 1. Because of its oxidation-reduction properties, laccase has found numerous applications in food, chemical processing, paper, the textile industry and environmental protection as well as in clinical analysis. Native and immobilized forms of these enzymes are used in biosensors and many biofuel cells [26]. Specifically in the case of laccase, copolymerization of laccase solutions has resulted in higher durability of their activity on the surface matrix [23] and relatively higher potential, which may be significant for its future usage [27]. To date, using wet chemistry, the most popular homobifunctional reagents for protein immobilization or copolymerization are: glutaraldehyde, diisocyanates and diisothiocyanates, homopolyfunctional epoxides; “zero-length crosslinking” with the use of carbodiimides (CDI), acyl azide, or dye mediated photo-oxidation [22,28,29,30]. These processes are lengthy and complex, adding to cost of production, so that copolymerization of laccase by the much simpler method of plasma treatment offers new perspectives for new practical applications.

## 2. Materials and Methods

Four experiments were carried out:

- **Experiment 1-Exposure of laccase solution in water to plasma.** A solution of laccase in water covering a glass slide was subjected to pure Helium Corona pin-to-plane jet plasma. The experimental factors were the applied voltage used for plasma generation and the time of the exposure of the solution to plasma. The experimental response was the laccase activity % relative to the non-plasma treated control solution.
- **Experiment 2-Plasma deposition of laccase onto water.** A laccase solution was injected into a Corona plasma jet directed onto a film of water lying on top of a glass slide. The experimental factors were, again, the applied voltage and the time of exposure to plasma. The response was laccase activity %.
- **Experiment 3-Plasma deposition of solid laccase coatings onto glass slides–optimization of plasma generation voltage.** Laccase solution was injected into a Corona plasma jet directed onto a glass slide. The experimental factor was the applied voltage. The response was laccase activity nkat/L.
- **Experiment 4-Plasma deposition of solid laccase coatings onto glass slides—optimization of Helium carrier gas flow rate.** Done as per Experiment No. 3. The experimental factor was the flow rate of Helium carrier gas and the response was laccase activity nkat/L.

Experimental and processed sample characterization procedures are now described in Section 2.1 to Section 2.11. Not all were used in all experiments.

### 2.1. Preparation of Solid Support or Matrix

The SPP process was applied in the form of a low energy density Helium Corona plasma jet to directly polymerize laccase molecules and either fire them into a water film on a glass slide or attach them as a coating onto the surface of clean glass slide supports. The condition and purity of the glass slide surface is critical to remove uncontrolled variables from the experimental conditions. Accordingly, a series of cleaning steps were applied to the glass slide to remove chemical and biological contaminants which would otherwise compromise both the coating adhesion and as-deposited film performance and analysis. Thus, the glass slides were immersed in a solution of water and ethyl alcohol (96%) in an ultrasonic bath for 30 s. After drying at 50 °C for 2 h, the glass slides were further cleaned by immersion for 10 min in low pressure plasma in a barrel-type system (Diener Electronic Zepto Plasma) using a conventional oxygen/argon gas mix. This type of process is well known for removing organic contaminants with extremely high efficacy and leaving glass surfaces atomically clean.

### 2.2. Laccase Source and its Purification

Laccase from *Cerrena unicolor* C-139 was obtained from the culture collection of the Regensburg University (Regensburg, Germany) and deposited in the fungal collection of the Department of Biochemistry, Maria Curie-Sklodowska University, Poland under the strain number 139. Laccase from the fermentor scale cultivation was obtained according to already reported procedures after ion exchange chromatography on DEAE Sepharose (fast flow) [33] and lyophilized on Labconco (Kansas City, MO, USA, FreeZone Lyophilizer) in vials. Enzyme activity was measured spectrophotometrically with syringaldazine as the substrate for laccase in 0.1 mol/dm^3^ Mc Ilvaine buffer, pH 5.5 [33]. The protein content was determined according to Bradford with bovine albumin as the standard [34] or at 280 nm using the Nanodrop ND-1000 spectrophotometer (NanoDrop Technol. Inc., Wilmington, DE, USA). The concentration of isolated and frozen (−18 °C) enzyme was C_laccase_ = 178 mg/cm^3^ and activity 186,000 nkat/dm^3^. After lyophilizing, the laccase activity dissolved in 1 mL of MilliQ water was 2,350,100 nkat/dm^3^ (141 U/cm^3^) per mg of protein. The stock solution (“Stock Solution”) of laccase was prepared by the dilution of laccase liophilizate in 1 mL of MilliQ water and split into 10 (2000 μL) Eppendorf tubes containing 100 μL solutions which were kept at −70 °C. For the preparation of working solutions, the Stock Solution was diluted by a 10% water solution of ethanol in a 1000 μL volume. This is called the “Standard Solution of Laccase”.

### 2.3. Laccase Dispensing into the Corona SPP System and Coating

**Figure 2:**
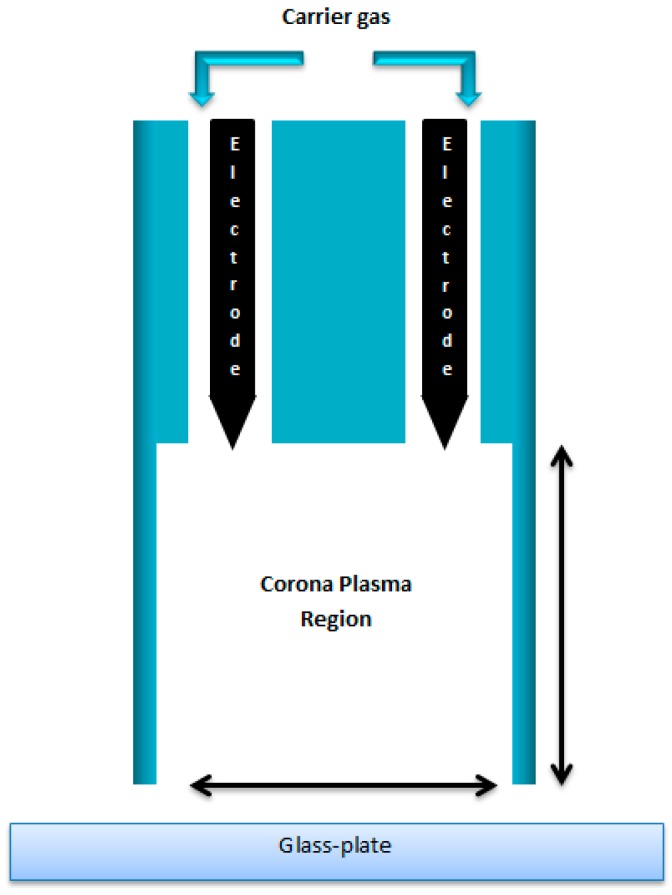
Schematic of the Corona plasma discharge device applied for deposition of laccase coatings.

In Experiments 2, 3 and 4 the Standard Solution of laccase was injected using a syringe pump (SyringinePomp, New Era Pump Systems, Inc., Farmingdale, NY, USA) at 200 µL/min into a nebulizer (Burgener Type 2100). The 10% of ethyl alcohol in the Standard Solution greatly reduces surface tension and increases the efficiency of atomization so that the “atomized” solution takes the form largely of vapor. The “atomized” vapor is injected by the nebulizer into and carried by a Helium gas stream into a plasma discharge zone generated by a Continuous Wave (CW) ~20 kHz AC high voltage (2–6 kV) from the points of two pin electrodes to create sprays of Corona plasma. The schematic of the device used for deposition of laccase coatings and for the exposure to plasma of laccase solution already on the glass slide in Experiment 1 is showed in Figure 2. In the case of Experiment 1, no laccase solution was injected into the plasma jet, which consisted only of the Helium gas stream. The coatings of Experiments 2–4 were deposited onto the cleaned glass slides (with water coating in the case of Experiment 2), which were moved at 1.67 mm/s in a single axis back and forth under the open end of the Corona jet apparatus.

**Figure 3:**
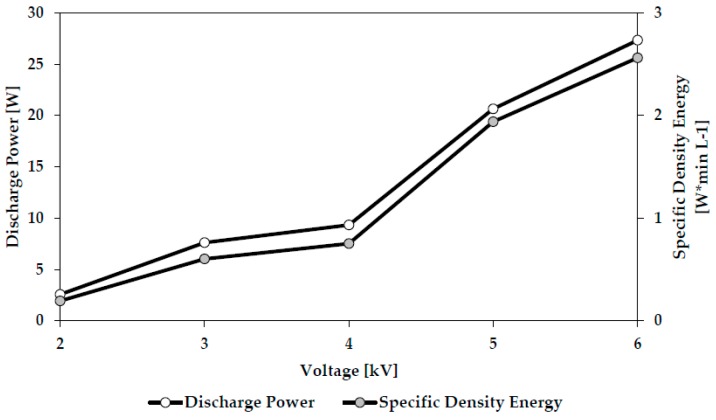
Plasma Discharge Power and Specific Energy Density vs. Applied Voltage used to generate the Helium Corona pin-to-plane plasma discharge of Figure 2.

Figure 3 shows the relationship between applied voltage (kV) and plasma discharge power (W) and between applied voltage and specific energy density (W × min/L) of the plasma for this system.

### 2.4. Water Wash

In Experiments 3 and 4 immediately following coating by the plasma jet, the sample slide was immersed without agitation in 1.5 mL of MilliQ deionized water in a Petri dish for 90 s. It was then removed and the procedure was repeated in a fresh Petri dish of water. The washed slide was removed and immediately proceded to the laccase activity determination of Section 2.5. The procedure was designed to remove from the coated substrate unpolymerized and unbonded materials.

### 2.5. Laccase Activity Determination

Laccase activity in the as-deposited material of Experiments 2, 3 and 4 was estimated with syringaldazine (Aldrich, St. Louis, MO, USA) as the substrate on a Helios γ spectrophotometer [34]. The as-deposited (Experiment 2) or the washed (Experiments 3 and 4) slides were transferred into a reaction mixture containing 0.1 M McIlvaine buffer, pH 5.5 with syringaldazine as substrate [34]. After 1 min incubation the supernatant absorption at 525 nm was determined. The Activity was expressed as nano katals per liter of culture medium (nkat/L). 

### 2.6. Polymerized Particle Size and Zeta Potential

The size of as-deposited polymerized laccase particles in Experiment 2 and the zeta potential were characterized using a ZetaSizer Nano ZS (Malvern Instr., Westborough, MA, USA) instrument working at an angle of 175° employing the Smoluchowski equation. The particle size was taken as the mean value of three measurements on each sample.

### 2.7. EDX Composition and AFM Micrographs

Coated glass slides were mounted on a sample holder in a field emission scanning electron microscope FEI Quanta 250 FEG equipped with EDS-EDAX energy measurement for elemental composition analysis of surfaces. The 3-D structure of the as-received biocoatings was determined using Atomic Force Microscopy (AFM) (Nanoscope, Veeco, Plainview, NY, USA). Analysis of the 3-dimensional structures was performed by WSxM 5.0 Develop 8.2 program [24].

### 2.8. Molecular Weight of Laccase Preparation Proteins and Their Profile

The proteins of laccase preparation were separated and quantitated by a lab-on-a-chip electrophoresis technique using the denaturated condition with SDS based on their molecular mass in comparison with the marker protein ladder [35,36]. The chip-based separations were performed using an Agilent 2100 bioanalyzer (Agilent Technologies, Santa Clara, CA, USA) in combination with the HS Protein 250 Plus lab chip kit and the dedicated Protein 250 software assay on 2100 expert software. Chips were prepared according to the protocol provided by the Protein 250 lab chip kit. The Protein 250 ladder (10–250 kDa) and the internal markers were used as reference for sizing and relative quantification.

### 2.9. Fourier Transform Infrared Spectroscopy (FTIR) Measurement

FTIR spectrum of laccase biocoating was collected using spectrophotometer NICOLET 380 FTIR (Thermo Scientific, Waltham, MA, USA) by deposition of laccase onto KBr plates containing 30% of powdered glass. 

### 2.10. Long-Term Stability of Laccase Biocoatings

Durability of laccase coating deposited using the Corona SPP method was studied by Electrochemical Analyzer EA-9 (MTM-ANKO, Warsaw, Poland) using the square-wave voltammetric method in a three-electrode system at room temperature. The system comprised a working electrode being a glassy carbon electrode coated with laccase deposited by Corona SPP, an Ag|AgCl (3M KCl) electrode as the reference electrode and a platinum electrode as an auxiliary electrode. The experiment recorded the current generated by enzymatic reaction with Rutin (at a constant concentration) which is directly proportional to the activity of the laccase biocoating [37]. The biocoating functionality of the immobilized laccase was determined daily by measuring the current in relative units defined as the ratio of the current measured each day to the output signal received on day 1 of the measurement series.

### 2.11. Circular Dichroism (CD) Spectroscopy of Laccase

Far-UV CD spectra (180–260 nm) of both untreated and plasma (U = 8kV, V_He_ = 10 L/min) treated laccase were recorded using Chirascan Plus (Applied Photophysics, Leatherhead, UK) at concentrations of 0.05 mg protein per 1 mL of water. The spectrum of water was used as a blank and subtracted from the average of ten spectra in order to obtain the spectrum of laccase.

## 3. Results & Discussions

### 3.1. Experiment 1-Exposure of Laccase Solution in Water to Plasma

**Figure 4:**
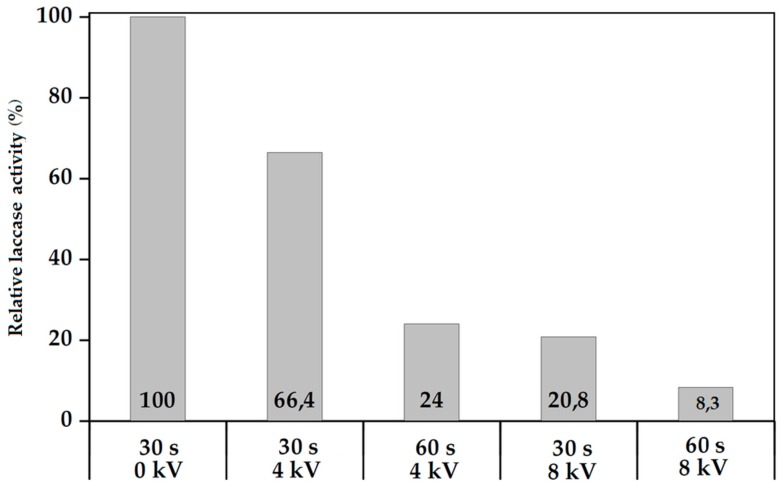
Experiment No. 1: Relative laccase activity (%) versus different applied voltages and plasma exposure times at constant V_He_ = 10 L/min.

The laccase Stock Solution (1 mL) was deposited onto a cleaned glass slide. The addition of alcohol into the Standard Solution which assists the atomization was not required in this case. The slide with its laccase solution covering was placed below the end of the Corona jet as per Figure 2 and exposed to a pure Helium Corona plasma for 30 or 60s at varying applied voltages and at a constant Helium flow rate of 10 L/min. Figure 4 displays the data:

The control sample (0 kV, 30 s) was not exposed to plasma. The bioactivity of the solution is increasingly degraded as the plasma increases in severity, namely in power density and duration of exposure. This is intuitively expected. However, it might also be expected that the water would afford more protection to the laccase molecules in solution, but apparently not in this configuration where the thickness of the water layer is small and the combination of Helium gas flow and plasma wind may be generating significant turbulence and churn in the water layer, thus readily and repeatedly exposing laccase molecules in solution to energetic and damaging plasma species. This data appears to confirm one component of plasma damage to a monomer undergoing plasma deposition, namely the damage caused by continued exposure to plasma after deposition of the monomer onto a surface even in the case of a potentially protective medium such as water. Again this seems intuitively obvious and consistent with other work but the extent of damage from this type of plasma jet appears significant and argues against extended exposure of coated substrates beyond achieving the minimum required level of coating durability and activity.

### 3.2. Experiment 2-Plasma Deposition of Laccase onto Water

**Figure 5:**
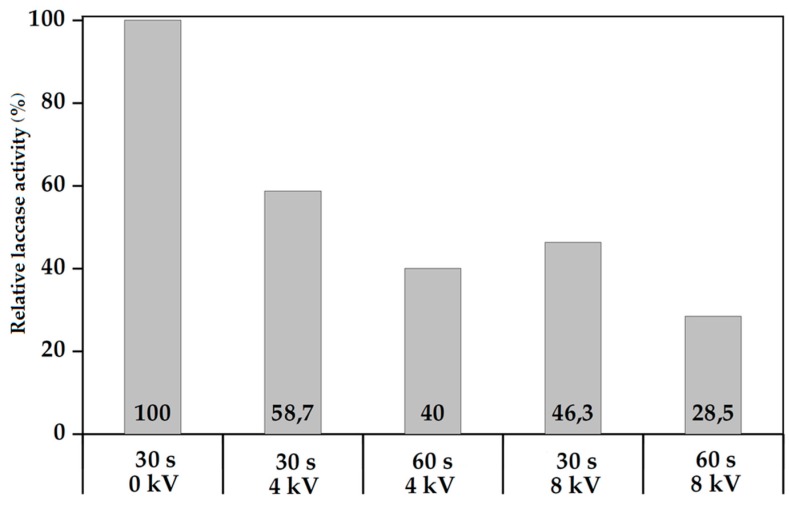
Experiment No. 2: Relative laccase activity (%) versus different applied voltages and plasma exposure times at constant V_He_ = 10 L/min. 60 s activities are divided by 2 to normalize.

Experiment 2 was prepared by placing 1 mL of clean, deionized water onto a cleaned glass slide and exposing this for 30 or 60 s to a Corona plasma, into which the nebulizer Standard Solution at 200 μL/min in the method of Section 2.3 was injected, thus exposing the water to the Corona SPP process output. Helium gas flow was constant at 10 L/min and the applied voltage was 4 or 8 kV. Control samples were generated at 0 kV, namely no plasma, to prepare a benchmark of “native”, untreated laccase whose activity was designated as 100%. The plasma processed preparations of laccase were compared to the control sample by determination of relative laccase activity. Molecular weight, particle size and Zeta potential, were also measured. Figure 5 displays the data which applies normalization by division by 2 of the 60 s activity data. Due to the process control parameters, with the exception of time, remaining constant over the two 4 kV experiments and again over the two 8 kV experiments, it seems reasonable to assume that the key dynamic responses, deposition rate, etch rate (if any) and rate of molecular damage should also remain constant throughout both time periods. Due to the very low ion energies here, molecular damage of the as-deposited coating is likely to affect only the top few monolayers so that each deposited layer will be quickly protected from further damage by the growing coating. Thus, it seems likely that relative laccase activities of the two coatings, 30 and 60 s, will only, to first order, be determined by process time so that normalization by dividing the 60 s activity by 2 seems appropriate.

We see plausible trends in the form of a monotonic reduction in laccase activity as the plasma treatment becomes harsher in both time and voltage. This clearly indicates the need to minimize exposure of the enzyme to plasma consistent only with the minimum required to achieve the necessary degree of cross-linking for adequate biofunctionality combined with coating durability. Deactivation of enzymes by room temperature plasma jet could be due to high energy electrons, ions, atomic or molecular radicals, heat or electric field [38]. The presence in the plasma jet of fast electrons can theoretically cause redox reactions of copper atoms included in the laccase molecule, which would decrease the activity of laccase.

Deactivation of laccase under the plasma influence is highly dependent on the specific values of the process control parameters and indicates the need for optimization of each process to produce the desired surface coating for a specific application, e.g., biocatalyzer. The obtained results however, confirm that a flow of laccase solution in atomized vapor form can pass through this Helium Corona plasma type whilst retaining a significant proportion of its biofunctionality.

Direct comparison with the data of Figure 4 seems impossible, as in this Experiment 2 total laccase volume is not exposed to plasma for the same integrated time as that of Experiment 1. Thus, it seems impossible from the data to decouple plasma damage effects in time of flight mode (from monomer injection to deposition) from monomer damage in situ, as deposited on the substrate. All we can state is that even this low energy density CW Helium Corona plasma type generates substantial (~50%) damage to the bioactive laccase molecular precursor volume in either or, more likely, both as-deposited and in-flight modes in-line with universal general experience over years of plasma deposition. Pulsing of this plasma type instead of CW generation may be an option to reduce this proportion of damage, as seen in low pressure plasma processes back in the 1990s [39].

### 3.3. Experiment 3-Plasma Deposition of Solid Laccase Coatings onto Glass Slides—Optimization of Plasma Generation Voltage

**Figure 6:**
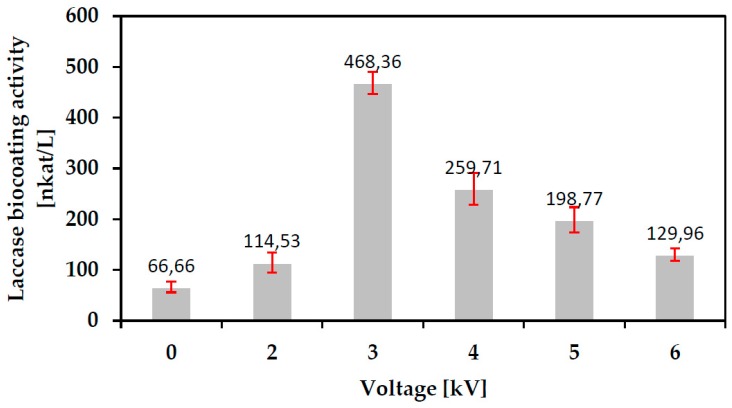
Laccase biocoating activity vs. Plasma generation applied voltage for as-deposited coatings on glass slides (V_He_ = 10 L/min).

Plasma deposition processes were carried out as per Section 2.3 onto cleaned glass slides. The coated slides were water washed as per Section 2.4. The process control parameter or factor to be optimized in this Corona SPP coating process development was the applied voltage to the electrodes, U (kV). The experimental response was the impact of the SPP process on the biological activity of the as-deposited coating as measured per Section 2.5 in nkat/L. Figure 6 shows the data:

For each factor (kV) value three depositions were carried out. The average activity is plotted with the uncertainty being the standard deviation of the three experiments for each data point.

The magnitude of the applied voltage will impact both the energy density (J mm^3^) as per Figure 3 and the number of reactive species (e.g., fast electrons, ions, radicals, photons) to which unit volume (mm^3^) of the laccase monomer is exposed both inside the plasma jet and on the glass surface. Application of low voltage values for plasma generation results in relatively low energy density and reactive species concentration inside the plasma jet. This seems likely to result in a relatively ineffective polymerization process and poor coating durability so that post-plasma washing may remove a high percentage of the coating resulting in low activity of the final washed slide. Application of high voltage values is likely to result in high energy density plasma causing molecular damage and fragmentation resulting in enzyme deactivation and denaturation consistent with the observed decreasing activity on the final slide. Thus, the data appears to reflect the influence of the two competing factors determining bioactivity of the final, washed coating, namely plasma damage to the monomer and the degree of cross-linking imparting coating durability.

### 3.4. Experiment 4-Plasma Deposition of Solid Laccase Coatings onto Glass Slides—Optimization of Helium Carrier Gas Flow Rate

**Figure 7:**
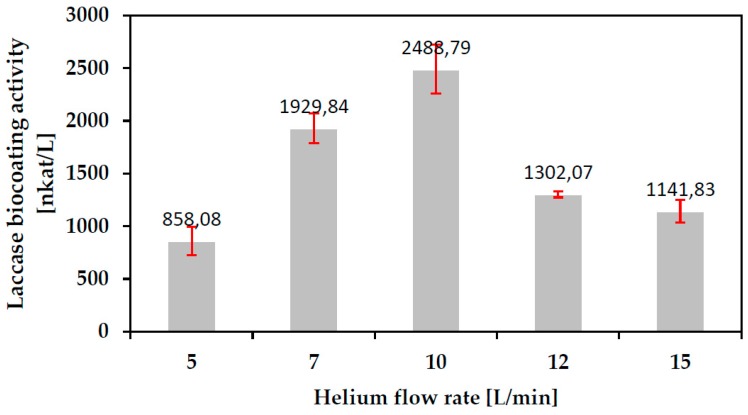
Laccase biocoating activity vs. Helium carrier gas flow rate for as-deposited coatings on glass slides (U = 3 kV).

Plasma deposition processes were again carried out as per Section 2.3 onto cleaned glass slides and the coated slides washed as per Section 2.4. The process control factor to be optimized in this Corona SPP coating process development was the flow rate of Helium carrier gas, V_He_ (L/min). The experimental response was the impact of the SPP process on the biological activity of the as-deposited coating as measured per 2.5 in nkat/L. Figure 7 shows the data:

The flow rate of Helium determines the residence time of unit volume of monomer in plasma. Thus the flow rate will, again, impact both the energy density and the number of reactive species to which unit volume of the laccase monomer is exposed. At low flow rates the extended residence time is likely to result in excessive damage to the laccase molecule in flight between injection and deposition, thus reducing coating activity. At high flow rates it is possible that exposure of unit volume of monomer to plasma energy and energetic species is insufficient for maximum polymerization so that much of the coating is washed away. Thus, the data again appears to reflect the influence of the two competing factors determining the bioactivity of the final, washed coating, namely plasma damage to the monomer and the degree of cross-linking imparting coating durability.

Biocoatings for further experiments (EDX, FTIR, AFM, long term stability) were deposited using Corona Discharge generated at the determined optimal values of U = 3 kV and V_He_ = 10 L/min.

### 3.5. Durability of Corona SPP Deposited Laccase Biocoating

**Figure 8:**
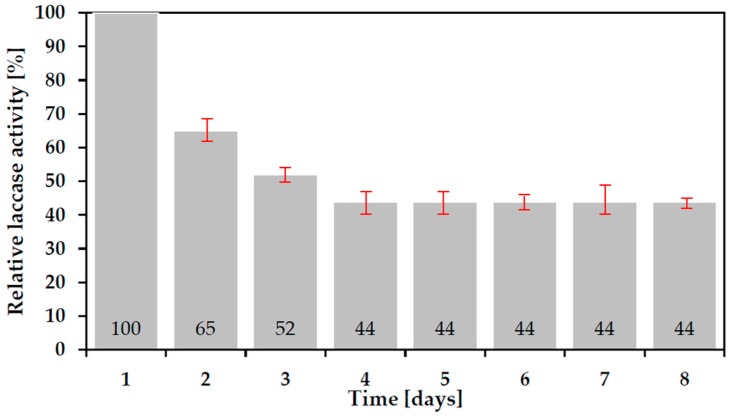
Durability of Corona SPP laccase coatings deposited on glass slide (U = 3 kV, VHe = 10 L/min, sample: rutin solution at concentration 0.0001 mol/dm^3^).

The durability of the as-deposited laccase biocoating is critical for performance in, for example, a bio-sensor role. The Relative Laccase Activities of three as-deposited coatings were measured by the electrochemical method of Section 2.10 over a period of 8 days. Figure 8 shows the average result obtained after subtraction of background values. As is seen, 56% of the activity is lost in the first four days, after which the activity stabilizes at 44% of the original. This may reflect a continuation of the washing out of the more loosely bonded species in the coating until only tightly bonded material remains.

### 3.6. Study of Plasma Polymerization Reactions

#### 3.6.1. Molecular Weight Distribution

**Figure 9:**
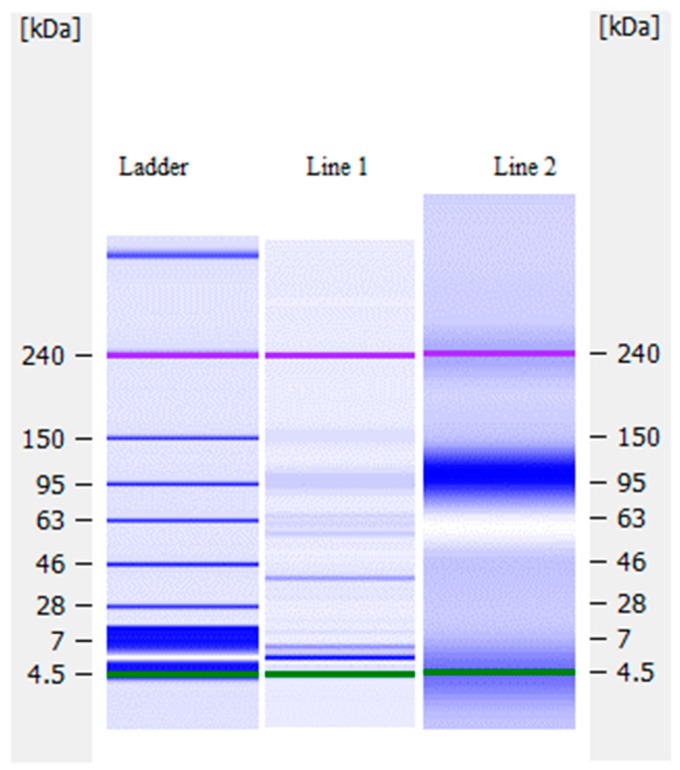
Tear protein profile of the Experiment 2 samples: Line 1 = ‘Native’ non-plasma treated laccase; Line 2 = Plasma deposited sample at 4 kV, 30 s.

As-deposited samples from the 4 kV, 30 s process of Experiment 2 (deposition into water) were analyzed for molecular weight distribution as described in Section 2.8. The results are shown in Figure 9.

The results showed both polymerization and fragmentation of laccase molecules after plasma treatment. The main laccase protein in the “native” non-plasma treated control showed the molecular weight to be about 40 kDa (Line 1) [39]. Degraded and fragmented protein molecules are seen in the plasma treated sample (Line 2) appearing in a wide band between 4 to 7 kDa. However, substantial polymerization of the monomer molecules is also seen after plasma treatment in the band around 240 kDa and in a wide band 70–140 kDa. It is suggested that the same analysis carried out on samples immobilized on glass as per experiments 3 and 4 may show weaker signals from the fragments in the range 4–7 kDa due to removal by the washing process.

#### 3.6.2. Particle Size Distribution

As-deposited samples from the 4 kV, 30 s process of Experiment 2 (deposition into water) were measured for Particle Size Distribution using the DLS (Dynamic Light Scattering) technique as per Section 2.6. The native, control untreated laccase was found to have a size distribution 70–120 nm (90% of molecules) with a mean characteristic size of 100 nm consistent with the literature. In the case of the plasma treated sample, the distribution shows 12% of the sample to be in the size band 40–70 nm (fragmentation) and 85% of the sample to be in the band 300–700 nm (polymerization). The size of 1000 nm has been seen in laccase polymerized by conventional processes such as hydrophobization [40]. From the distribution of sizes, it is noteworthy that the effect of the plasma on treated samples is to produce more polymerization of the laccase than fragmentation of the molecule although the possibility of multi-stage processes in which the molecule is firstly fragmented and then polymerized cannot be ruled out.

#### 3.6.3. Zeta Potential

As-deposited samples from the 4 kV, 30 s process of Experiment 2 (deposition into water) were measured for Zeta potentials per Section 2.6. Zeta potential changes confirm the polymerization process during plasma deposition by reducing the number of positively charged moieties within the precursor structure. Zeta potential changes thus indicate the strength of the plasma polymerization process of the biological precursor. All plasma modified laccase solutions exhibited higher negative potentials (−7.42 and −4.57 mV) in comparison to the untreated native material (−3.72 mV). It can be speculated that the differences in potentials come from the lower number of free Lysine residues in the case of the plasma modified samples, thus there was an observed decrease in the number of positively charged moieties while the number of negatively charged residues did not change [41].

#### 3.6.4. Circular Dichroism

In order to study structural changes in laccase due to plasma treatment, a CD spectrum was investigated. The literature [42,43,44,45] indicates that atmospheric pressure plasmas cause differences in the structures of lysozyme, polyphenoloxidase, peroxidase, alkaline phosphatase and myoglobin protein. Table 1 shows changes in the secondary structure of laccase enzyme resulting from treatment with Helium Corona Plasma Jet generated at U = 8 kV and V_He_ = 10 L/min. The aim of this study was to assess loss of laccase biological activity as a result of structural changes. Therefore, laccase was treated here by Corona Discharge at the highest voltage in order to cause the highest molecular damage and decrease in activity (Figure 4 and Figure 5). The data in Table 1 suggest that the decrease in laccase activity may come from partial transformation of its antiparallel β 2D structure into α-helix structure, caused by interaction of the enzyme with active plasma species present in the Corona discharge such as He ions or radicals generated by degradation of –COOH, –NH2, and –CONH groups. Also potentially present in the discharge are hydroxyl radicals (OH^•^) and superoxide anion radicals (O_2_^−^). The literature [44,46,47] indicates that such radicals can react with amino acids such as cysteine, aromatic rings of phenyloaniline, tyrosine and tryptophan, causing a reduction in alkaline phosphatase activity by the degradation of aromatic rings [44]. The crystallographic structure of laccase shows that its active center contains histidines bonded with Cu atoms. Degradation of histidine imidazole rings by active plasma species can cause a reduction in enzyme activity. It is also possible that some leakage of the ambient air surrounding the Corona discharge into the plasma region or close to the substrate surface could generate ozone molecules (O_3_). Reference [48] indicates that O_3_ molecules can be responsible for increasing the α-helix substructure content in lyzosyme 3D models after plasma treatment. Such a mechanism may also be taking place in these experiments with *C. unicolor* laccase.

### 3.7. Structural and Composition Studies of Coatings

#### 3.7.1. Energy Dispersive X-ray Analysis

**Figure 10:**
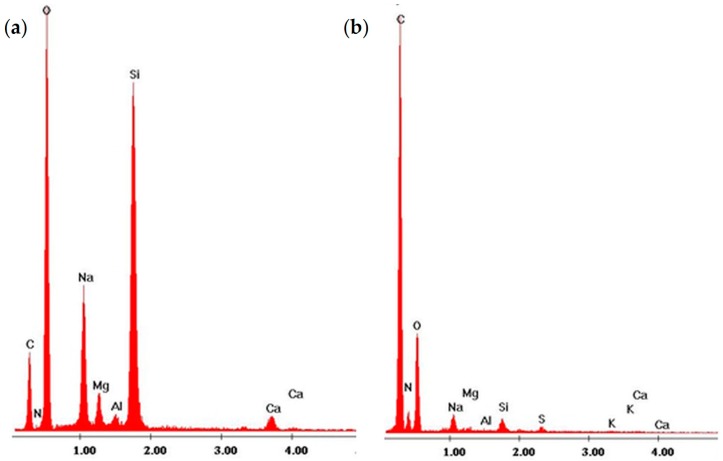
The elemental composition of glass support surface (**a**) before plasma and (**b**) after plasma deposition of laccase.

The post-wash plasma polymerized coatings obtained in the Corona SPP plasma jet generated at U = 3 kV and V_He_ = 10 L/min were studied by Energy-Dispersive X-ray Spectroscopy elemental analysis per Section 2.7 as shown in Figure 10.

The presence of laccase biomolecules deposited on the glass by plasma was indicated by the elemental spectrum of the coating, as shown in Figure 10b. The spectrum shows elevated amounts of Carbon, Oxygen, Sulphur and Nitrogen, the basic components of the amino acids forming the polypeptide chain of laccase and the carbohydrate component parts of this glycoprotein. These peaks are absent or much lower on spectra obtained for clean glass slides (Figure 10a). Spectra of clean glass slides include high peaks from glass oxides such as sodium, magnesium and silicon.

#### 3.7.2. Atomic Force Microscopy

**Figure 11:**
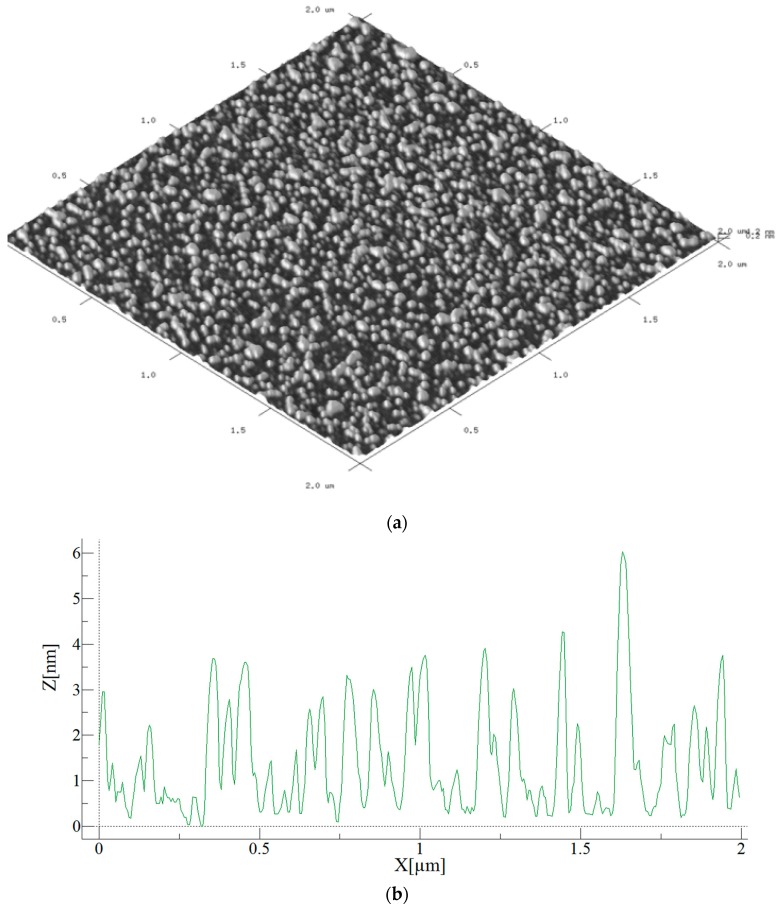
(**a**) AFM picture of laccase plasma coated mica substrate, and (**b**) AFM cross-section of the laccase coating.

Atomic Force Microscopy (AFM) was applied to image the as-deposited coatings as per Section 2.7 and Figure 11. These coatings were deposited on Mica, as the technique does not respond well to a glass substrate. Figure 11a shows the three-dimensional structure of the as-deposited laccase coating. Figure 11b is a cross section of the above surface. The zero of the Z thickness parameter corresponds to the naked, uncoated glass substrate. Over the small sample characterized it is seen that the coating is of only a few nm in thickness.

#### 3.7.3. Fourier Transform Infra-Red Spectroscopy

**Figure 12:**
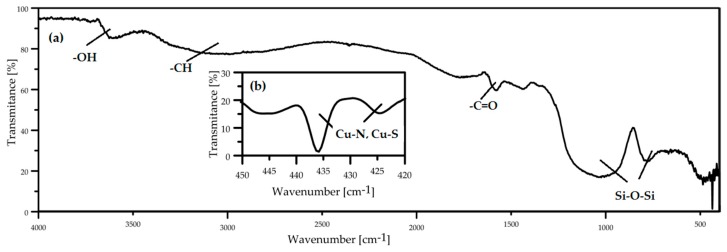
FTIR spectrum of laccase biocoating in range of wavenumbers from 4000 to 400 cm^−1^ (**a**) and 420 to 450 cm^−1^ (**b**).

FTIR measurement was carried out to identify chemical bonds in the laccase biocoating on a glass slide. The FTIR spectra (Figure 12) show peaks originating from the glass slide and from the laccase biomolecule. The two wide peaks at ~1100 and ~800 cm^−1^ originate from Si–O–Si bonds of the glass [49]. Also present are peaks at ~3600, ~3200 and 1600 cm^−1^ corresponding to –OH,  –CH and –C=O functional groups of the laccase molecule [31]. Oxygen functional groups seen in the spectrum are consistent with the O peak seen in the EDX chemical composition spectra of Figure 10. For a biologically active coating the most important peaks appear at ~475 and ~425 cm^−1^, corresponding to Cu–N and Cu–S bonds [50] which, as is seen in the Figure 1 molecular structure, occur in the active center of laccase. Their presence confirms that deposition of the biocoating by Corona Plasma Jet generated at 3 kV allows retention of the active center of the molecule and, thus, the biological activity of the precursor.

### 3.8. Mechanisms of Corona SPP Laccase Biocoating Deposition

The mechanism of laccase biocoating formation by Corona SPP is complex and almost certainly involves both cross-linking of laccase molecules in the plasma jet region as well as such cross-linking at the surface of the growing film. In addition, at the initial stage of deposition, reactions between the laccase and the glass substrate are needed to achieve bonding of the coating to glass. Cross-linking between laccase molecules probably occurs through radical reactions leading to amide bond formation. Bonding of the laccase coating to glass may take place through three parallel mechanisms: electrostatic interactions, adsorption of laccase by the glass surface and formation of hydrogen bonds with the glass.

To evaluate the possible mechanisms of biocoating formation, we assumed that only external amino acids in the laccase enzyme structure take part in cross-linking and bonding reactions. This allows retention in our model of the molecule’s active center structure in unchanged form. On the basis of this assumption we selected the seven amino acids appearing in the external regions of the laccase molecule as potentially able to play a role in cross-linking and bonding reactions, namely arginine (ARG), aspartic acid (ASP), glutamine (GLN), serine (SER), threonine (THR), tyrosine (TYR) and lysine (LYS). This selection was done on the basis of the laccase crystallographic structure analysis obtained from Protein Data Base (PDB ID: 3DIV) by visualization using the Chimera (Version 1.1, RBVI, 2004) software. Examples of important groups in cross-linking and bonding are identified by the modeling as amine groups in ARG, ASP, LYS and GLN, carboxyl groups in ASP, carbonyl groups in GLN and hydroxyl groups in SER, LYS, THR and TYR, the latter of which can interact with the glass molecule to deliver bonding.

The structures modeled indicate that regions can be identified in these amino acids: (I) the regions responsible for creation of amide bonds between amine and carboxyl groups in the amino acids resulting in cross-linking between laccase molecules and (II) the regions responsible for bonding with the surface of the glass substrate. For example, in the case of arginine the regions I can be seen as the single bonded-to-carbon oxygen atom of the carboxyl group with its attached hydrogen atom and the nitrogen atoms of the molecule’s amine groups. The O–H are removed from the carbon C4 and replaced by one of the nitrogen atoms from the amine groups of another arginine or other amino acid molecule, thus forming the cross-linking amide bond. One amine group hydrogen atom will also be removed from the molecule and combined with the liberated O–H from the carboxyl group to form water. Regions II are regions of electrostatic charge with either a positive or negative imbalance. Such charge concentrations are likely to give rise to electrostatic interactions between glass and amino acid molecules, perhaps based on induction of local charges on the glass slides. Interactions between these opposite charges create bonds between protein and the glass surface, causing the initial monolayers of the deposited laccase to adhere to the substrate. Additionally, it is likely that the plasma plays a significant role in both cleaning the substrate surface and creating active, high energy sites in both glass and laccase molecule through bombardment with energetic plasma species.

## 4. Conclusions

Conventional pin-to-point Corona plasma discharge was successfully used in SPP processes to immobilize into water and onto glass polymerized bioactive coatings of the oxidoreductase enzyme protein. Such coatings showed reduced bioactivity relative to the ‘native’ untreated enzyme, undoubtedly due to a degree of damage to and fragmentation of monomer molecules by the active, energetic species in the plasma such as fast electrons, ions, radicals and photons. However, as much as 59% of relative laccase activity (Figure 5) was retained by the optimum plasma deposition into water process and plasma deposition onto glass delivered 7 times the laccase activity of the same non-plasma deposition process in the coating after water washing (Figure 6). This latter result would seem to be due to the ability of the plasma to both crosslink the monomer and more strongly bond it to the glass surface by a combination of surface cleaning and activation of both glass and laccase. FTIR analysis indicated that the core copper containing moieties at the center of the molecule largely remain undamaged by this plasma type so that bonding and cross-linking reactions are likely to involve mainly species around the outer perimeter of the molecule. Formation of amide bonds between amino acids are likely to play an important role in the cross-linking reactions between laccase molecules and that amino acid molecular electrostatic charge distributions, perhaps aided by exposure to plasma, may account, at least in part, for electrostatic bonding of laccase to glass. The plasma deposited coating was found to retain 44% of its activity over a period of 8 days despite daily washing and testing. The combination of these coating performance parameter values for retained activity and durability under water wash indicates that a relatively simple Corona SPP plasma process for deposition of biocoatings may offer enhanced solutions for biocatalyst or sensor and lab-on-a-chip applications.

## Figures and Tables

**Table 1:** The main 2D substructures in laccase molecules before and after plasma treatment.

	Percentage Content [%]	
	Non-Plasma Treated Laccase	Plasma Treated Laccase (U = 8 kV, V_He_ = 10 L/min, t = 60 s)
Helix	6.3	12.7
Antiparallel	47.9	32.2
Parallel	3.7	4.5
Beta-turn	16.8	20.3
Rndm. Coil	29.3	32.3
